# Measuring cortical mean diffusivity to assess early microstructural cortical change in presymptomatic familial Alzheimer’s disease

**DOI:** 10.1186/s13195-020-00679-2

**Published:** 2020-09-17

**Authors:** Philip S. J. Weston, Teresa Poole, Jennifer M. Nicholas, Nicolas Toussaint, Ivor J. A. Simpson, Marc Modat, Natalie S. Ryan, Yuying Liang, Martin N. Rossor, Jonathan M. Schott, Sebastien Ourselin, Hui Zhang, Nick C. Fox

**Affiliations:** 1 Dementia Research Centre, UCL Institute of Neurology, Queen Square, Box 16, London, WC1N 3BG UK; 2 Department of Medical Statistics, London School of Hygiene & Tropical Medicine, London, UK; 3 Transitional Imaging Group, Centre for Medical Image Computing, University College London, London, UK; 4 Department of Biomedical Engineering & Imaging Sciences, King’s College London, London, UK; 5 Microstructure Imaging Group, Centre for Medical Image Computing, University College London, London, UK

**Keywords:** Alzheimer’s disease, Familial, Autosomal dominant, Presymptomatic, MRI, Diffusion, Mean diffusivity, Cerebral cortex

## Abstract

**Background:**

There is increasing interest in improving understanding of the timing and nature of early neurodegeneration in Alzheimer’s disease (AD) and developing methods to measure this in vivo. Autosomal dominant familial Alzheimer’s disease (FAD) provides the opportunity for investigation of presymptomatic change. We assessed early microstructural breakdown of cortical grey matter in FAD with diffusion-weighted MRI.

**Methods:**

Diffusion-weighted and T1-weighed MRI were acquired in 38 FAD mutation carriers (17 symptomatic, 21 presymptomatic) and 39 controls. Mean diffusivity (MD) was calculated for six cortical regions previously identified as being particularly vulnerable to FAD-related neurodegeneration. Linear regression compared MD between symptomatic and presymptomatic carriers and controls, adjusting for age and sex. Spearman coefficients assessed associations between cortical MD and cortical thickness. Spearman coefficients also assessed associations between cortical MD and estimated years to/from onset (EYO). Across mutation carriers, linear regression assessed associations between MD and EYO, adjusting for cortical thickness.

**Results:**

Compared with controls, cortical MD was higher in symptomatic mutation carriers (mean ± SD CDR = 0.88 ± 0.39) for all six regions (*p* < 0.001). In late presymptomatic carriers (within 8.1 years of predicted symptom onset), MD was higher in the precuneus (*p* = 0.04) and inferior parietal cortex (*p* = 0.003) compared with controls. Across all presymptomatic carriers, MD in the precuneus correlated with EYO (*p* = 0.04). Across all mutation carriers, there was strong evidence (*p* < 0.001) of association between MD and cortical thickness in all regions except entorhinal cortex. After adjusting for cortical thickness, there remained an association (*p* < 0.05) in mutation carriers between MD and EYO in all regions except entorhinal cortex.

**Conclusions:**

Cortical MD measurement detects microstructural breakdown in presymptomatic FAD and correlates with proximity to symptom onset independently of cortical thickness. Cortical MD may thus be a feasible biomarker of early AD-related neurodegeneration, offering additional/complementary information to conventional MRI measures.

## Background

In Alzheimer’s disease (AD), it has become increasingly important to understand the timing and nature of neurodegeneration and to develop sensitive methods for its detection and tracking. Of particular interest are the changes that characterise very early disease prior to the onset of clinical symptoms, as targeting treatments at this stage, before significant irreversible neuronal loss, may provide the greatest chance of success [1].

Autosomal dominant familial AD (FAD) shares many features—pathologically, radiologically and clinically—with the more common sporadic form of disease [2]. Unlike sporadic disease, FAD mutation carriers have relatively predictable ages at symptom onset based on family history [3], facilitating the prospective study of asymptomatic individuals prior to cognitive decline.

Early neurodegeneration involving cortical grey matter is a recognised feature of both sporadic and familial AD, with structural MRI identifying similar characteristic patterns of macrostructural loss within the cerebral cortex [4, 5]. Such cortical thinning predates symptom onset.

Diffusion-weighted imaging (DWI) allows assessment of changes at the microscopic level, with neuronal loss and the breakdown of microstructural barriers, such as myelin, cell membranes and intracellular organelles, resulting in a measureable difference in the diffusion of water molecules [6]. Measurement of such changes may provide additional and/or complementary information to conventional T_1_-weighted imaging, with studies suggesting that DWI changes may be more predictive of early progressive cognitive change than macrostructural atrophy [7, 8]. While most DWI studies in AD have focused on white matter [7, 9–11], it would follow, given the known early involvement of cortical grey matter [12], that measurement of microstructural DWI changes in the cortex could prove to be valuable in detecting and better characterising early neuronal breakdown. Grey matter changes have been found to be more closely related to clinical decline than those in white matter [13].

In AD, DWI measures of cortical grey matter have been found to differ between groups of individuals with amnestic MCI who do and do not go on to progress to AD dementia [8]. However, the ability of DWI to detect presymptomatic cortical changes, and their association with proximity to symptom onset, remains uncertain. It is also unclear whether cortical DWI metrics provide any additional information over and above that given by measurement of cortical thickness.

We used DWI to measure mean diffusivity (MD) in a group of FAD mutation carriers and non-carrier controls. MD is a DWI metric that increases with microstructural breakdown. It assesses diffusion in all directions and is therefore particularly suited to assessing grey matter, where diffusion is isotropic [14]. We hypothesised that cortical MD increases presymptomatically in FAD and correlates with proximity to symptom onset. Moreover, we aimed to assess the relationship between cortical MD and cortical thickness and whether measuring microstructural change with cortical MD provides additional information above and beyond cortical thickness alone.

## Methods

### Participants

Seventy-seven participants were recruited to a cohort study of FAD at the Dementia Research Centre, University College London: 38 with FAD mutations in either the presenilin 1 or amyloid precursor protein genes and 39 healthy controls. Of the mutation carriers, 17 had progressive cognitive symptoms and 21 were presymptomatic. All participants underwent clinical assessment, including a semi-structured interview, neurological examination and completion of the Clinical Dementia Rating scale (CDR) [15]. Estimated years to/from symptom onset (EYO) was calculated for the mutation carriers by subtracting the age at which their parent first developed progressive cognitive symptoms from the participant’s age. Blood samples were collected from all FAD family members, with mutation status assessed using Sanger sequencing.

### MRI acquisition

All participants were scanned on the same 3-Tesla Siemens TIM Trio scanner using a 32-channel phased array head-coil. A sagittal 3D MP-RAGE T1-weighted volumetric MRI (echo time/repetition time/inversion time = 2.9/2200/900 ms, dimensions of 256 × 256 × 208, voxel size of 1.1 mm isotropic) was acquired. Two 64-direction DWI sequences were acquired with a single shot, spin-echo echo planar imaging (EPI) sequence (field of view 240 × 240 mm; matrix 96 × 96; yielding a voxel size of 2.5 mm isotropic; 55 contiguous axial slices; repetition time 6800 ms; echo time 91 ms; *b* value 1000s/mm^2^). We acquired nine acquisitions without diffusion weighting (*b* = 0 s/mm^2^).

### Image analysis

Cortical parcellation was performed using FreeSurfer v5.30 (http://surfer.nmr.mgh.harvard.edu). The diffusion-weighted images were registered to the first *b* = 0 image using NiftyReg [16] and corrected for susceptibility [17], motion and eddy current distortion. The two DWI acquisitions for each participant were combined to increase the signal-to-noise ratio, with tensor fitting performed with a single tensor model using NiftyFit [18] to produce MD maps.

**Fig. 1:**
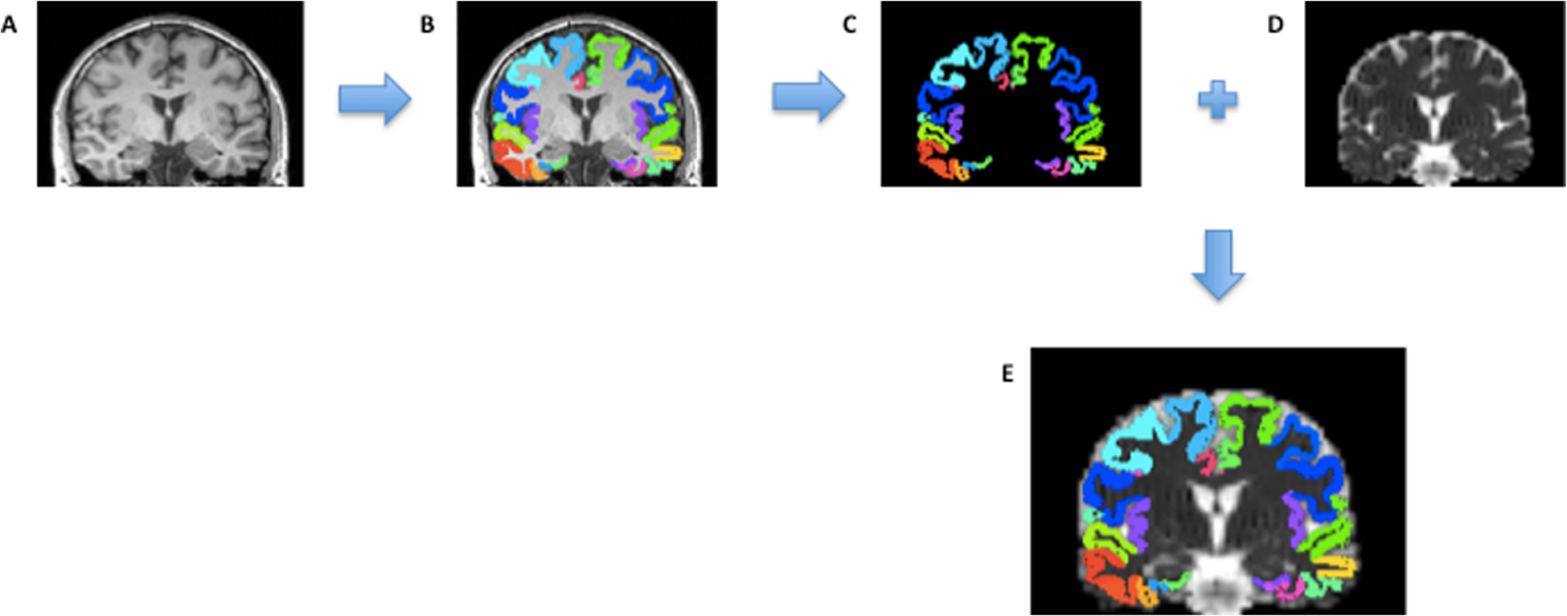
Cortical parcellation and measurement of cortical MD. Each T1 image (**a**) underwent cortical parcellation using FreeSurfer (v5.30) (**b**). The FreeSurfer label for each ROI was warped into diffusion space (**c**) and registered to the MD map (**d**) to allow extraction of mean MD within that region (**e**)

We restricted our analysis to six cortical regions of interest (ROIs) identified previously as comprising the FAD cortical signature [5]: entorhinal cortex, inferior parietal cortex, precuneus, superior frontal cortex, superior parietal cortex and supramarginal cortex. The FreeSurfer label (derived from the T_1_-weighted acquisition) for each cortical ROI was warped from T_1_ to diffusion space and registered to the MD map, to allow extraction of mean MD within that region (Fig. 1). To reduce potential CSF-grey matter partial volume effects when calculating regional MD, a weighted mean was calculated. Weights were derived from interpolation of the T1 space binarised label map, which differentiated cortical grey matter from non-cortex (i.e. white matter or CSF), towards the lower resolution diffusion space. Cortical thickness for each region was calculated in FreeSurfer using the same procedure as described previously [5].

### Statistical methods

The presymptomatic mutation carriers were split at the median EYO (− 8.1 years) into early presymptomatic (early PS, i.e. more than 8.1 years before predicted onset) and late presymptomatic (late PS, i.e. 8.1 years or less before predicted onset), resulting in a total of four subgroups: controls; early PS; late PS; and symptomatic. Cortical MD was averaged across left and right hemispheres. For each ROI, a single linear regression was used to compare MD between the four subgroups, adjusting for age and sex. If a global test provided evidence of a main effect of group, then post hoc pairwise comparisons between sub-groups were carried out. Homoskedasticity and normality assumptions concerning residuals were checked and were not materially violated.

Given non-normal distributions and non-linear relationships, non-parametric Spearman correlation coefficients were used to assess the unadjusted association between EYO and cortical MD in each of the ROIs, first across all mutation carriers and then in presymptomatic carriers only. Spearman coefficients were also calculated to assess the association in mutation carriers between cortical MD and cortical thickness.

Additionally, in order to investigate whether MD provides additional information above cortical thickness, in mutation carriers, linear regression assessed associations between MD and EYO, after adjusting for cortical thickness. Evidence for a quadratic relationship with EYO was investigated using Wald tests. For each region, graphs were plotted to show estimated MD against EYO for a person with the study sample’s mean cortical thickness for that region. Assumptions concerning residuals were checked and were not materially violated.

All analyses used Stata v15 (StataCorp, College Station, TX, USA). No adjustments were made for multiple testing [19].

## Results

Mutation carriers and controls were well matched for age (42.6 years [SD 9.5] vs 44.6 [9.5]) and sex (15 male/24 female vs 18 male/20 female). The group demographics, with mutation carriers split between symptomatic, early PS and late PS, are shown in Table 1.

**Table 1:** Participant demographics

*N*	Controls	Early presymptomatic	Late presymptomatic	Symptomatic
	39	10	11	17
Age, years (mean (SD))	44.6 (9.5)	34.8 (5.5)*	41.9 (8.4)	47.6 (9.0)
Gender, m/f	15/24	3/7	5/6	10/7
EYO, years (mean (SD))	–	− 11.8 (2.6)	− 3.3 (4.6)	4.8 (4.0)^^^
Global CDR (mean (SD))	0	0	0	0.85 (0.39)**

For comparison of age, unadjusted linear regression was used; for comparison of CDR, Fisher’s exact test was used

*EYO* estimated years to onset, *CDR* Clinical Dementia Rating scale

*Evidence of a difference (*p* < 0.05) compared with controls

**Evidence of a difference (*p* < 0.001) compared with controls

^^^2 missing values

**Fig. 2:**
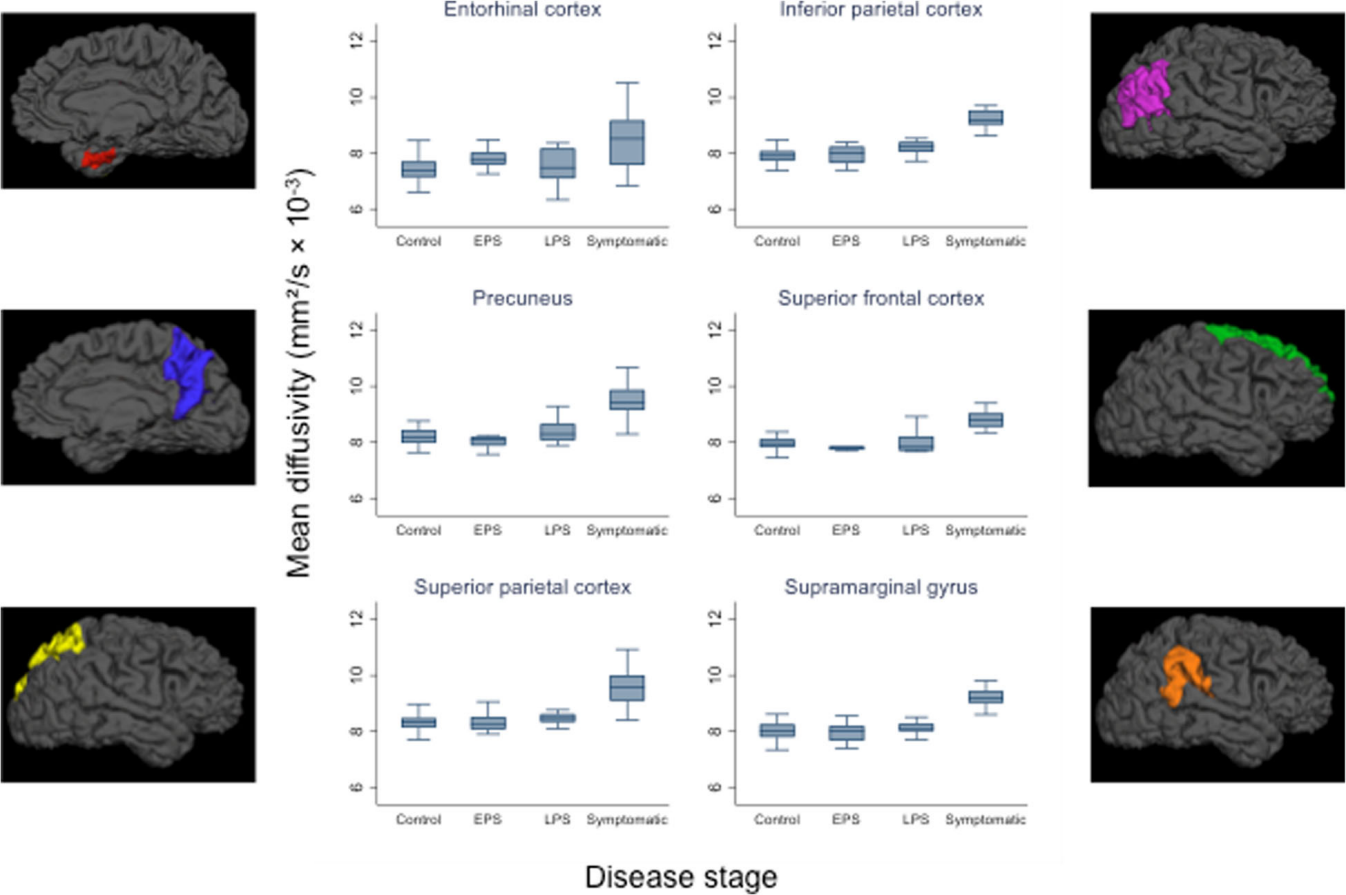
Box plots of cortical MD in the six regions of interest across the different disease stages. EPS, early presymptomatic; LPS, late presymptomatic

For each ROI, there was strong evidence (*p* < 0.0001) for a main effect of group, so post hoc pairwise comparisons were carried out. After adjusting for age and sex, symptomatic individuals had significantly higher MD compared to controls, and to both presymptomatic groups, in all six cortical regions (*p* < 0.00001 for all, except in entorhinal cortex when compared with early PS (*p* = 0.03) and with late PS (0.0006)) (Fig. 2, Table 2, Additional file 1). Cortical MD was higher in the late PS group compared with controls across all six regions, although this reached statistical significance only in the inferior parietal cortex (estimated adjusted difference in means 0.34 mm^2^/s × 10^−3^, 95% CI 0.12, 0.57; *p* = 0.003) and the precuneus (0.28 mm^2^/s × 10^−3^, 0.01, 0.55, *p* = 0.04). No statistically significant differences in MD were found between the early PS group and controls.

**Table 2:** MD values

	Controls	Early presymptomatic	Late presymptomatic	Symptomatic
Entorhinal cortex MD (SD)	7.43 (0.52)	7.73 (0.46)	7.50 (0.64)	8.49 (1.03)**
Inferior parietal cortex MD (SD)	7.89 (0.24)	7.93 (0.36)	8.19 (0.26)*	9.26 (0.55)**
Precuneus MD (SD)	8.18 (0.28)	8.07 (0.33)	8.43 (0.42)*	9.50 (0.62)**
Superior frontal cortex MD (SD)	7.98 (0.28)	7.84 (0.22)	7.99 (0.37)	8.89 (0.57)**
Superior parietal cortex MD (SD)	8.34 (0.35)	8.32 (0.35)	8.52 (0.36)	9.58 (0.66)**
Supramarginal cortex MD (SD)	8.04 (0.36)	7.95 (0.36)	8.15 (0.31)	9.24 (0.62)**

Observed mean (SD) MD values (mm^2^/s × 10^−3^) are shown for each cortical ROI across the four groups. For comparison of MD, linear regression was used, adjusted for age and sex

*MD* mean diffusivity

*Evidence of a difference (*p* < 0.05) compared with controls

**Evidence of a difference (*p* < 0.001) compared with controls

When analysing the MD values, one outlier was identified (and re-checking the images found no reason to omit these data). Re-running the regression analyses without the outlier did not cause any meaningful change to the results.

Across mutation carriers (*n* = 36 as EYO was missing for two symptomatic participants), a positive correlation was found between cortical MD and EYO (*p* < 0.0001) in all regions except entorhinal cortex (*p* = 0.14), with MD progressively increasing over time. When including presymptomatic individuals only, the association between MD and EYO only reached formal statistical significance in the precuneus (Spearman’s rho = 0.46, *p* = 0.04) but a non-significant positive association was still seen in four of the five other regions. The one exception was, again, the entorhinal cortex, for which during the presymptomatic period the association between cortical MD and EYO was estimated to go in the opposite direction (Table 3).

**Table 3:** Association between MD and EYO in presymptomatic mutation carriers

Region	Correlation with EYO (Spearman’s rho)	***p*** value
Entorhinal cortex	− 0.43	0.05
Inferior parietal cortex	0.28	0.23
Precuneus	0.46	0.04
Superior frontal cortex	0.40	0.07
Superior parietal cortex	0.32	0.16
Supramarginal cortex	0.11	0.63

Spearman correlation coefficients and *p* values for the association between MD and EYO in presymptomatic mutation carriers only, in each of the six cortical regions of interest

*EYO* estimated years to onset

**Fig. 3:**
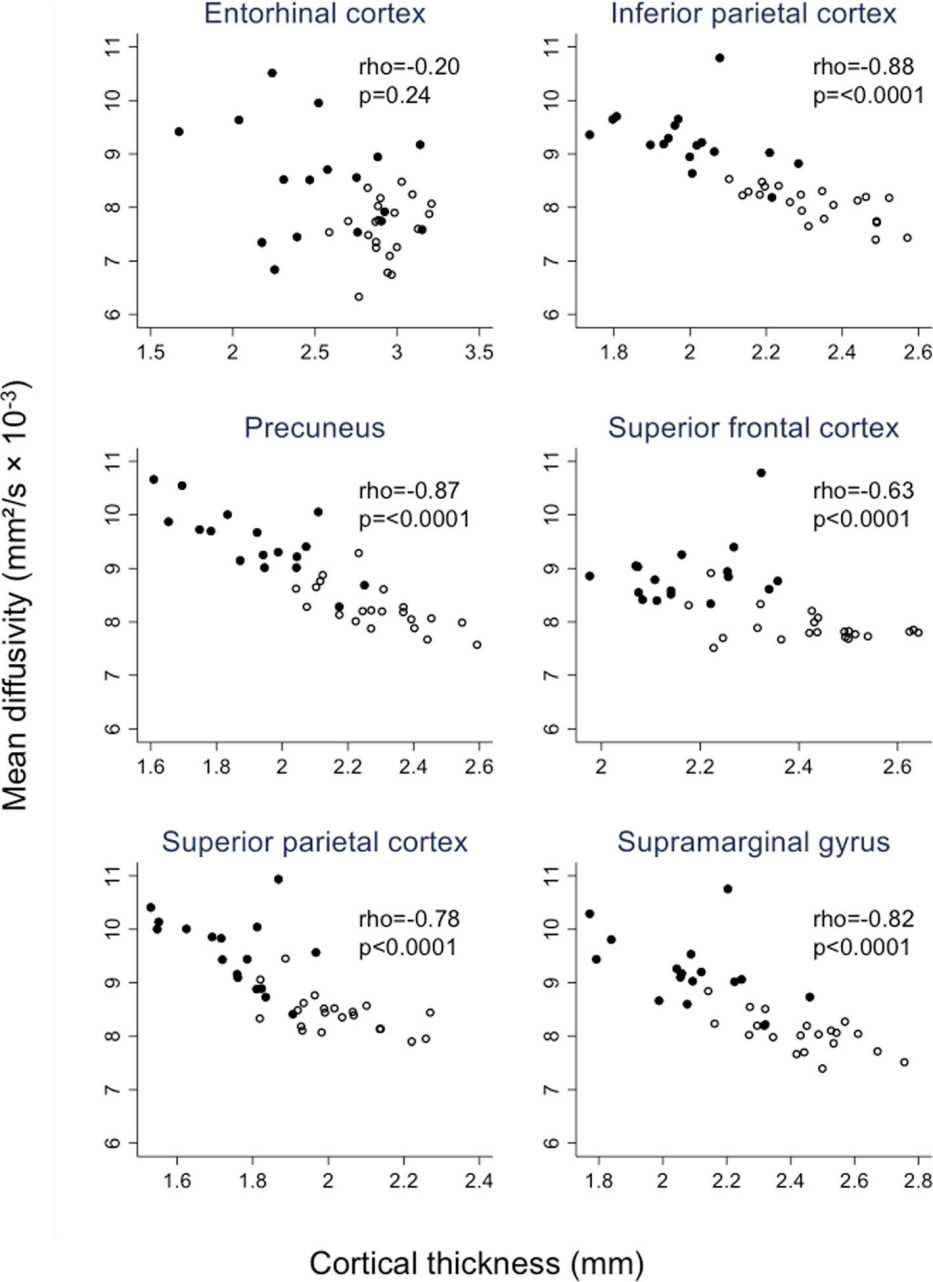
Association between cortical mean diffusivity and cortical thickness. Scatter plots are shown, with accompanying Spearman correlation coefficients, for the association between mean diffusivity and cortical thickness across mutation carriers. Black dots represent symptomatic carriers and white dots presymptomatic carriers

In mutation carriers (*n* = 38, no missing values), cortical MD demonstrated a significant negative Spearman correlation with cortical thickness (*p* < 0.0001) in all ROIs except entorhinal cortex (*p* = 0.24) (Fig. 3). A similar negative association was found in non-carriers (rho <− 0.6, *p* ≤ 0.0005) for all regions except entorhinal cortex and superior frontal cortex.

**Fig. 4:**
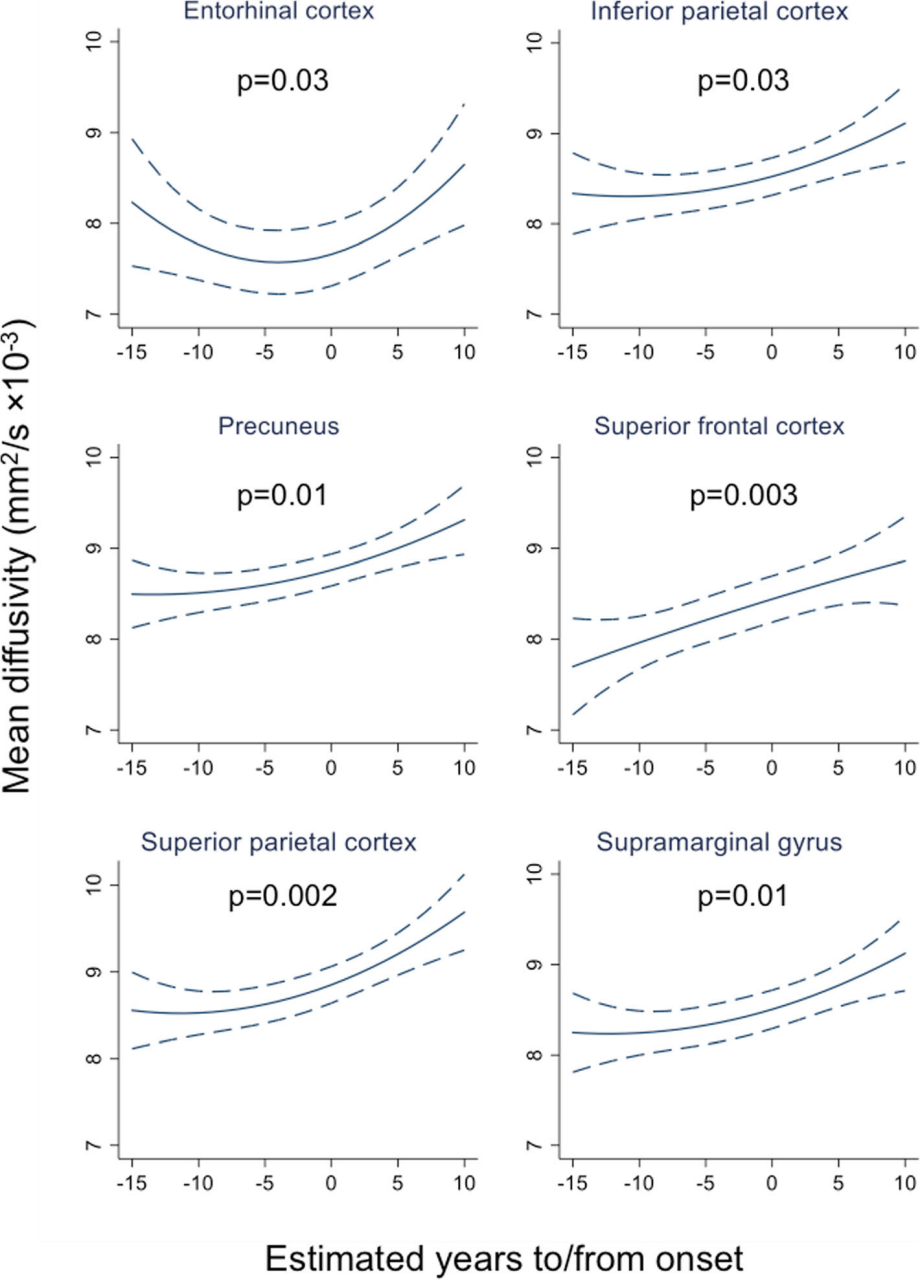
Relationship between mean diffusivity and estimated years to/from symptom onset in mutation carriers. Graphs show linear regression of MD against EYO after adjusting for cortical thickness and including a quadratic term for EYO in all models for consistency. For each region, cortical thickness was set at the mean value for the study sample for that region: entorhinal (2.75 mm), inferior parietal (2.20 mm), precuneus (2.14 mm), superior frontal (2.32 mm), superior parietal (1.91 mm) and supramarginal (2.30 mm). The *p* value is from a test of the association between MD and EYO after adjusting for cortical thickness. MD, mean diffusivity; EYO, estimated years to/from symptom onset

Across mutation carriers (*n* = 36), a linear regression of cortical MD against EYO showed that for all six cortical regions there remained a significant association (*p* < 0.05) between MD and proximity to symptom onset after adjusting for cortical thickness (Fig. 4), with MD estimated to increase over time for individuals with the same cortical thickness. Removal of the outlier previously identified led to slightly weaker (*p* < 0.10) evidence of an association for three regions (entorhinal, inferior parietal and precuneus), with the other three (superior frontal, superior parietal, supramarginal) remaining as *p* < 0.05.

## Discussion

We found evidence that late presymptomatic FAD mutation carriers (i.e. within 8.1 years of predicted symptom onset) have significantly increased cortical MD compared with controls in both the precuneus and the inferior parietal cortex. Furthermore, among mutation carriers, cortical MD was associated with proximity to/from expected symptom onset, independent of cortical thickness.

It has previously been shown that cortical diffusivity differs between AD dementia and controls and between progressive versus non-progressive MCI [8, 20]. Here, we also show that MD is higher in those with symptomatic AD compared to healthy individuals, in a group of symptomatic familial AD individuals who on average were only mildly clinically affected (mean CDR = 0.85). Furthermore, we showed, albeit with limited statistical significance, that cortical diffusivity changes were identifiable in presymptomatic FAD a number of years before the onset of cognitive symptoms, suggesting presymptomatic microstructural cortical breakdown.

Of the six ROIs, the most significant presymptomatic MD changes were seen in the inferior parietal cortex and the precuneus. Both of these regions have been identified as being affected early in AD [4, 5, 21], with DWI studies in symptomatic AD showing that measurement of precuneus diffusivity allows differentiation between AD-MCI and controls and between AD and dementia with Lewy bodies [22, 23]. While in five of the six ROIs cortical MD was positively correlated with EYO, it was only in the precuneus that we found a significant association to persist after removing the symptomatic individuals and focusing on the presymptomatic participants only. This suggests MD in the precuneus may closely reflect disease activity in the period approaching the onset of clinical symptoms.

One previous smaller study also investigated cortical diffusivity change in presymptomatic FAD [24]. However, while they too found presymptomatic changes in the precuneus, they observed a fall in MD rather than an increase, which was felt possibly to be due to a presymptomatic inflammatory process [25]. A recent small positron emission tomography study using a tracer for brain astrocytosis appears to support the possibility of such a relationship, with astrocytic inflammation decreasing as cortical diffusivity increased [26]. A further study that investigated cortical MD in presymptomatic sporadic AD (diagnosed based on measurement of CSF Aβ) reported a biphasic distribution, with those who had Aβ pathology but no evidence of neurodegeneration having reduced MD, but those with both Aβ pathology and neurodegeneration having increased MD [27]. Interestingly, however, an increase in MD in the precuneus and inferior parietal cortex—the two regions in our study to show the earliest rise—did not occur until after symptom onset. Here, in the majority of regions assessed, we found no evidence to support an initial MD reduction. The MD increase we observed across multiple regions in the late presymptomatic group, and the positive association with increasing EYO, likely indicate freer diffusion of water molecules as cellular barriers break down, consistent with what one may expect during a neurodegenerative process.

The one region in our study in which MD did not show significant correlation with disease progression across the disease spectrum was entorhinal cortex. For this region, when looking in presymptomatic individuals only, the association went in the opposite direction to all other regions, with MD progressively decreasing with increasing proximity to symptom onset. There was greater inter-individual variability in MD in the entorhinal cortex than in other regions (Fig. 2), suggesting one explanation for the discrepancy being that the small size and the anatomical location of the entorhinal cortex, which can make it more difficult to parcellate accurately, may mean its MD signal is particularly vulnerable to CSF contamination. However, it may also be that, for the presymptomatic period covered by our study, the entorhinal MD does genuinely behave differently to other regions assessed. This possibility is reflected in the linear regression model of entorhinal cortex MD as a function of EYO (adjusted for cortical thickness), which shows MD in this region initially reducing before later progressively increasing (Fig. 4). Such an initial reduction in entorhinal cortex MD would be consistent with a previous finding from two other anatomically related regions—the hippocampus and cingulum—that have also been shown to have early restriction of diffusion [28].

We found a close association between cortical MD and cortical thickness in mutation carriers for the majority of the regions assessed, with increasing cortical MD being associated with decreasing cortical thickness. Cortical thickness measurement using FreeSurfer is a widely used and well-validated method of assessing macrostructural cortical change. The association between thickness and MD therefore provides further face-validity for cortical MD being a marker of cortical integrity/degeneration, with both reduction in thickness and increasing MD likely being part of the same pathological continuum. The fact that an association was also present in healthy ageing non-carriers further supports the idea of both cortical MD and cortical thickness measuring the same general characteristic, i.e. neuronal integrity/degeneration (albeit different aspects and/or stages), which is known to progressively alter, although to a lesser extent, in healthy ageing as well as neurodegenerative disease [29, 30]. However, importantly, we found that when it comes to association with disease stage or proximity to symptom onset (as determined by EYO), cortical MD shows an association even after adjusting for cortical thickness. This suggests that, rather than providing directly analogous information to that gained from cortical thickness alone, the microscopic changes detected by measuring MD provide independent information that is of additional value and may reflect early disease-related neuronal loss. This finding is consistent with a previous study of AD that assessed whole brain cortical MD and cortical volume, which found that while cortical MD showed a trend along the trajectory from normal controls to MCI to established AD dementia, cortical volume did not demonstrate the same pattern [8]. It is also consistent with findings from another neurodegenerative disease—frontotemporal dementia—where it has been found that differences between patients and controls were greater for cortical MD than for cortical thickness, with cortical MD also showing a closer association with other measures of disease severity [31]. However, it is difficult to say at present whether the association between MD and EYO being independent of cortical thickness means MD tells us something qualitatively different about what is happening from a neurodegenerative perspective or whether it is simply a more sensitive marker of neurodegeneration. Microstructural changes such as MD may lie upstream to macrostructural thickness changes [9, 32], and so could provide an earlier measure of change. Further studies, ideally with longitudinal assessment, will be required to confirm this.

## Limitations

This study has a number of limitations. The sample size was limited by the relative rarity of FAD, although to our knowledge, this is the largest DWI study of FAD to date. As each group represents a different disease stage, their average ages differed; however, we adjusted for age in the group comparisons. Although steps were taken to minimise the effects of partial volume, with our data (not presented here) showing that the correction made led to a significant reduction in partial volume effects, this cannot be eliminated completely. Additionally, while parental age at onset has been shown to correlate closely with actual age at onset, it remains a proxy measure [3]. Finally, the current study includes cross-sectional data only, meaning that it is not possible directly to assess association between MD and disease progression; assessment in future longitudinal studies of whether early microstructural loss as measured by MD is associated with rate of subsequent decline will be valuable.

## Conclusions

We have demonstrated that measurement of cortical MD is able to detect presymptomatic microstructural breakdown of the cerebral cortex in FAD and is associated with proximity to symptom onset independently of cortical thickness. Cortical MD may therefore provide complementary information to macrostructural atrophy. Our findings support the further investigation of cortical MD, particularly within the precuneus and inferior parietal cortex, as a potential marker of early AD neurodegeneration.

## Supplementary information

**Additional file 1.**

## Data Availability

The datasets used and/or analysed during the current study are available from the corresponding author on reasonable request.

## Abbreviations

AD: Alzheimer’s disease
FAD: Familial Alzheimer’s disease
MRI: Magnetic resonance imaging
MD: Mean diffusivity
EYO: Estimated years to/from onset
DWI: Diffusion-weighted imaging
MCI: Mild cognitive impairment
CDR: Clinical Dementia Rating scale
ROI: Region of interest
CSF: Cerebrospinal fluid
PS: Presymptomatic
