# Supplementary material for: Measuring cortical mean diffusivity to assess early microstructural cortical change in presymptomatic familial Alzheimer’s disease
Source: Alzheimers Res Ther. 2020 Sep 17;12:112. doi: 10.1186/s13195-020-00679-2 (PMC7499910; doi:10.1186/s13195-020-00679-2)
Supplement: Supplementary file 1 — Additional file 1. [file 13195_2020_679_MOESM1_ESM.docx]

**Supplementary Material**

**Table S1. Estimated difference in mean cortical MD (95% CI; p-value) (mm^2^/s ×10^-3^), after adjusting for age and sex**

Results are from linear regression models, adjusting for age and gender. Abbreviations: MD=mean diffusivity; Early PS = early presymptomatic; Late PS = late presymptomatic.

|  | **Adjusted difference in MD**  **(mm^2^/s ×10^-3^)** | **95% CI** | **p-value** |
| --- | --- | --- | --- |
| **Entorhinal cortex** |  |  |  |
| Early PS vs Controls | 0.41 | -0.09, 0.91 | 0.11 |
| Late PS vs Controls | 0.12 | -0.34, 0.58 | 0.60 |
| Symptomatic vs Controls | 1.07 | 0.68, 1.47 | <0.00001 |
| Late PS vs Early PS | -0.29 | -0.89, 0.30 | 0.33 |
| Symptomatic vs Early PS | 0.66 | 0.08, 1.24 | 0.03 |
| Symptomatic vs Late PS | 0.95 | 0.43, 1.48 | 0.0006 |
|  | | | |
| **Inferiorparietal cortex** |  |  |  |
| Early PS vs Controls | 0.18 | -0.07, 0.43 | 0.15 |
| Late PS vs Controls | 0.34 | 0.12, 0.57 | 0.003 |
| Symptomatic vs Controls | 1.33 | 1.14, 1.53 | <0.00001 |
| Late PS vs Early PS | 0.16 | -0.13, 0.45 | 0.28 |
| Symptomatic vs Early PS | 1.15 | 0.87, 1.44 | <0.00001 |
| Symptomatic vs Late PS | 0.99 | 0.73, 1.25 | <0.00001 |
|  | | | |
| **Precuneus** |  |  |  |
| Early PS vs Controls | 0.02 | -0.28, 0.31 | 0.91 |
| Late PS vs Controls | 0.28 | 0.01, 0.55 | 0.04 |
| Symptomatic vs Controls | 1.28 | 1.05, 1.51 | <0.00001 |
| Late PS vs Early PS | 0.27 | -0.08, 0.62 | 0.13 |
| Symptomatic vs Early PS | 1.26 | 0.92, 1.61 | <0.00001 |
| Symptomatic vs Late PS | 0.99 | 0.68, 1.31 | <0.00001 |
|  | | | |
| **Superior frontal cortex** |  |  |  |
| Early PS vs Controls | -0.06 | -0.33, 0.22 | 0.68 |
| Late PS vs Controls | 0.03 | -0.22, 0.28 | 0.79 |
| Symptomatic vs Controls | 0.89 | 0.68, 1.11 | <0.00001 |
| Late PS vs Early PS | 0.09 | -0.24, 0.42 | 0.58 |
| Symptomatic vs Early PS | 0.95 | 0.63, 1.27 | <0.00001 |
| Symptomatic vs Late PS | 0.86 | 0.57, 1.15 | <0.00001 |
|  | | | |
| **Superior parietal cortex** |  |  |  |
| Early PS vs Controls | 0.11 | -0.21, 0.43 | 0.51 |
| Late PS vs Controls | 0.21 | -0.08, 0.51 | 0.15 |
| Symptomatic vs Controls | 1.20 | 0.95, 1.46 | <0.00001 |
| Late PS vs Early PS | 0.11 | -0.27, 0.49 | 0.58 |
| Symptomatic vs Early PS | 1.10 | 0.72, 1.47 | <0.00001 |
| Symptomatic vs Late PS | 0.99 | 0.65, 1.33 | <0.00001 |
|  | | | |
| **Supramarginal cortex** |  |  |  |
| Early PS vs Controls | 0.04 | -0.27, 0.35 | 0.78 |
| Late PS vs Controls | 0.15 | -0.14, 0.43 | 0.31 |
| Symptomatic vs Controls | 1.15 | 0.91, 1.40 | <0.00001 |
| Late PS vs Early PS | 0.10 | -0.27, 0.47 | 0.58 |
| Symptomatic vs Early PS | 1.11 | 0.75, 1.47 | <0.00001 |
| Symptomatic vs Late PS | 1.01 | 0.68, 1.33 | <0.00001 |
